# Complete Genome Sequence of a Novel Chimpanzee Polyomavirus from a Western Common Chimpanzee

**DOI:** 10.1128/genomeA.01406-15

**Published:** 2016-01-21

**Authors:** Jolanda van Persie, Hester Buitendijk, Zahra Fagrouch, Willy Bogers, Tom Haaksma, Ivanela Kondova, Ernst J. Verschoor

**Affiliations:** a Department of Virology, Biomedical Primate Research Centre, Rijswijk, The Netherlands; b Animal Science Department, Division of Pathology and Microbiology, Biomedical Primate Research Centre Rijswijk, The Netherlands

## Abstract

We report here the full-length genome sequence of a novel chimpanzee polyomavirus. Viral sequences were recovered from colon, bladder, and ureter tissue from a western common chimpanzee. The virus is genetically closely related to the human BK polyomavirus.

## GENOME ANNOUNCEMENT

Polyomaviruses (PyV) are small DNA viruses that infect a wide range of mammals, birds, and fish. Since the discovery of the macaque polyomavirus SV40 in 1960, PyVs have been characterized from a range of nonhuman primates, including New World monkeys, Old World monkeys, and apes.

Here, we analyzed tissue samples from an individual belonging to the western chimpanzee subspecies (*Pan troglodytes verus*). In tissue samples from this animal, Ch-Regina, we previously detected a PyV (1), which belongs to the newly described PyV clade VI (2). Using clade-specific primer sets, we reanalyzed the tissue samples from Ch-Regina. Two more polyomaviruses were detected in this animal. In the colon and stomach cardia, we detected a clade III polyomavirus. Analysis of a 998-bp PCR fragment revealed 99% identity to PtrosPyV2, a chimpanzee PyV isolated from the eastern subspecies, *Pan troglodytes schweinfurthii* (3). A 518-bp PCR fragment was obtained using clade II-specific PCR primers in colon, urine, bladder, and ureter samples. Sequence analysis revealed 88% identity with the human BK virus. We amplified the complete genome, allowing an in-depth analysis of this new chimpanzee PyV. The virus has a genome of 5,163 bp and has a typical polyomavirus architecture, including an open reading frame encoding an agnoprotein. A BLAST search revealed that this virus, provisionally named *P. troglodytes verus* polyomavirus 8 (PtrovPyV8), has the highest identity (88%) to the recently described *Pan troglodytes troglodytes* polyomavirus 1 (PtrotPyV1) (4), and it has 81% identity with the human BK polyomavirus. The genetic relatedness of two viruses from different chimpanzee subspecies and the human BK virus is of interest, and one may speculate on a cospeciation between primate polyomaviruses and their hosts.

The human BK virus persists in the renourinary tract, and BK virus (BKV) has been detected in epithelial tissues of the ureter and bladder (5). A similar tissue distribution was found for PtrovPyV8, in addition to its presence in colon tissue. The presence of PtrovPyV8 in the colon and its related PtrotPyV1 in feces, is remarkable, as BK virus is regularly detected in colorectal tumors (6). This finding may point to a shared renourinary and colonic tropism for clade II polyomaviruses.

### Nucleotide sequence accession number. 

The complete genome of PtrovPyV8 has been deposited in GenBank under the accession number KT884050.

## References

[1] Deuzing I, Fagrouch Z, Groenewoud MJ, Niphuis H, Kondova I, Bogers W, Verschoor EJ 2010 Detection and characterization of two chimpanzee polyomavirus genotypes from different subspecies. Virol J 7:347. doi:10.1186/1743-422X-7-347. 21110837 PMC3003640

[2] Ehlers B, Moens U 2014 Genome analysis of non-human primate polyomaviruses. Infect Genet Evol 26:283–294. doi:10.1016/j.meegid.2014.05.030. 24933462

[3] Scuda N, Madinda NF, Akoua-Koffi C, Adjogoua EV, Wevers D, Hofmann J, Cameron KN, Leendertz SAJ, Couacy-Hymann E, Robbins M, Boesch C, Jarvis MA, Moens U, Mugisha L, Calvignac-Spencer S, Leendertz FH, Ehlers B 2013 Novel polyomaviruses of nonhuman primates: genetic and serological predictors for the existence of multiple unknown polyomaviruses within the human population. PLoS Pathog 9:e1003429. doi:10.1371/journal.ppat.1003429. 23818846 PMC3688531

[4] Madinda NF, Robbins MM, Boesch C, Leendertz FH, Ehlers B, Calvignac-Spencer S 2015 Genome sequence of a central chimpanzee-associated polyomavirus related to BK and JC polyomaviruses, *Pan troglodytes troglodytes* Polyomavirus 1. Genome Announc 3(5):e00888-15. doi:10.1128/genomeA.00888-15. 26337874 PMC4559723

[5] Hirsch HH, Steiger J 2003 Polyomavirus BK. Lancet Infect Dis 3:611–623. doi:10.1016/S1473-3099(03)00770-9. 14522260

[6] Casini B, Borgese L, Del Nonno F, Galati G, Izzo L, Caputo M, Perrone Donnorso R, Castelli M, Risuleo G, Visca P 2005 Presence and incidence of DNA sequences of human polyomaviruses BKV and JCV in colorectal tumor tissues. Anticancer Res 25:1079–1085. 15868949

